# Clinical Tear Fluid Proteomics—A Novel Tool in Glaucoma Research

**DOI:** 10.3390/ijms23158136

**Published:** 2022-07-23

**Authors:** Janika Nättinen, Ulla Aapola, Praveena Nukareddy, Hannu Uusitalo

**Affiliations:** 1 Eye and Vision Research, Faculty of Medicine and Health Technology, Tampere University, 33520 Tampere, Finland; ulla.aapola@tuni.fi (U.A.); praveena.nukareddy@tuni.fi (P.N.); hannu.uusitalo@tuni.fi (H.U.); 2 Tays Eye Centre, Tampere University Hospital, 33520 Tampere, Finland

**Keywords:** glaucoma, proteomics, tear fluid

## Abstract

Tear fluid forms the outermost layer of the ocular surface and its characteristics and composition have been connected to various ocular surface diseases. As tear proteomics enables the non-invasive investigation of protein levels in the tear fluid, it has become an increasingly popular approach in ocular surface and systemic disease studies. Glaucoma, which is a set of multifactorial diseases affecting mainly the optic nerve and retinal ganglion cells, has also been studied using tear proteomics. In this condition, the complete set of pathophysiological changes occurring in the eye is not yet fully understood, and biomarkers for early diagnosis and accurate treatment selection are needed. More in-depth analyses of glaucoma tear proteomics have started to emerge only more recently with the implementation of LC-MS/MS and other modern technologies. The aim of this review was to examine the published data of the tear protein changes occurring during glaucoma, its topical treatment, and surgical interventions.

## 1. Introduction

Tear fluid is a dynamic and complex fluid forming the outermost layer of the ocular surface. It is an optimal sample material for proteomics research for many reasons; it contains over 1500 measurable proteins [1,2,3], it can be collected in a non-invasive manner, and it is replenished with every blink of an eye.

**Figure 1:**
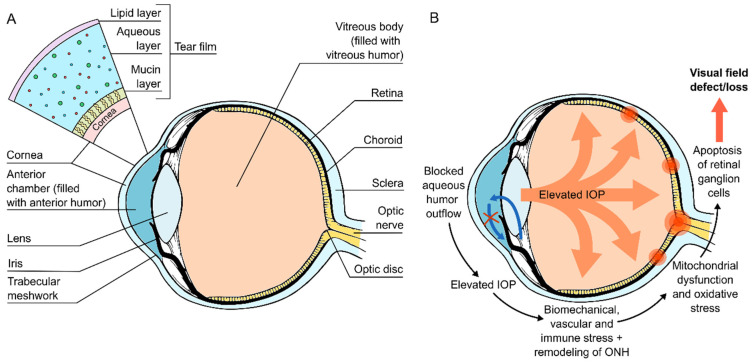
(**A**) Cross section of the eye, description of the tear film structure, and (**B**) development of glaucomatous changes in the eye. IOP, intraocular pressure; ONH, optic nerve head.

The tear fluid is commonly considered to consist of three layers: mucin, aqueous, and lipid layers (Figure 1A). The innermost mucin layer and its secreted and membrane-bound mucins make the tear film hydrophilic and connect the tear film to the ocular surface epithelium, providing more even tear fluid distribution. In addition to water, electrolytes, and metabolites, the aqueous layer in the middle contains most of the proteins carried to and from the ocular surface epithelium. These proteins in the aqueous layer make up the majority of the proteins identified in tear fluid, although the mucins from the mucin layer can also be detected. The outermost lipid layer, which can be studied with tear lipidomics [4], “seals” the tear film and prevents its evaporation from the ocular surface in-between blinks.

The tear fluid provides protection, nutrients, and oxygen to the underlying avascular tissue and removes waste from the ocular surface, thereby reflecting the health, disease, and recovery of the eye in many ocular diseases [5,6]. Several ocular surface diseases (OSDs), such as dry eye disease (DE) [7], Meibomian gland dysfunction (MGD) [8,9], conjunctivitis [10], and other inflammatory diseases have been studied by examining the protein and lipid changes in the tear fluid. In addition to diseases affecting the ocular surface directly, the tear protein changes in several systemic diseases such as Parkinson’s disease, cancer, and Alzheimer’s disease have also been studied previously [11]. In comparison to serum and plasma, tear fluid is a less complex body fluid for the analysis of systemic diseases, making it an appealing alternative for researchers searching for disease biomarkers.

As tear fluid can be used to study both ocular surface-affecting diseases as well as systemic diseases, it is no surprise that glaucoma, which is a collection of multifactorial diseases of the eye, is also being studied this way. Glaucoma is known to cause degenerative damage to the optic nerve and the loss of retinal ganglion cells (RGCs) (Figure 1B), resulting in gradual vision loss if left untreated, making it one of the most common reasons for irreversible blindness worldwide [12,13]. Prior to visual field loss, glaucoma is often initially characterized by an increased intraocular pressure (IOP) of over 21 mmHg. The IOP increase is usually the result of a blocked trabecular meshwork, which in return causes lowered outflow of the aqueous humor.

The most common forms of glaucoma are open-angle glaucoma (OAG), normal tension glaucoma (NTG), and angle-closure glaucoma (ACG). The glaucoma subtypes can be further categorized based on the pathological causes into primary and secondary (due to, e.g., trauma, disease, or exfoliation), based on the age of the patient into adult, juvenile, and congenital forms, and based on the disease progression into chronic and acute forms. The wide selection of subtypes makes glaucoma research more challenging and currently, many studies focus on the most common forms of glaucoma [14].

The presence, severity, and progression of glaucoma are currently established through clinical examinations. In addition to IOP measurement, the visual field, nerve fiber layer of the retina, optic disc, anterior chamber angle, and other alterations of the eye structures are examined by an ophthalmology expert [15]. Glaucoma is in most cases initially a symptomless disease, and thus the glaucomatous changes can develop unnoticed for years before diagnosis. So far, there are no treatments that can reverse the effects of glaucoma. For these reasons, effective diagnostic and treatment tools are needed to identify and treat individuals in the early stages or at risk of developing glaucoma.

Currently, the pathophysiological changes of the different glaucoma subtypes as well as the effects of glaucoma treatment are being studied using a broad selection of research methods. In the genomics field, clinical researchers are trying to identify the genetic risk factors affecting glaucoma development [16,17,18]. In clinical proteomics, lipidomics, and metabolomics fields, the pathogenesis of glaucoma and its therapeutic options are being studied using various sample materials such as tear fluid, aqueous humor, vitreous body, and serum [19,20,21]. This review focuses specifically on how clinical tear proteomics is used to examine the development and treatment of glaucoma.

## 2. Tear Fluid Analytics

Tear fluid samples can be taken in various ways, but Schirmer strips and capillary tubes are the two most common methods currently in use. In relation to tear proteomics, the most notable difference between these two sampling methods lies mainly in the protein types and protein numbers generated. The Schirmer strip, which is a filter paper placed under the lower eyelid, produces a higher number of quantified proteins and proportionally a higher number of proteins of an intracellular origin, whereas capillary tube samples generate proportionally more secreted and extracellular proteins [22]. There is also discussion on whether these two sampling methods are comparable in tear types, or whether the Schirmer strip induces more frequent reflex tearing due to the irritation of the ocular surface [23]. The disadvantages of capillary sampling include the small sample amount and more demanding sampling [24]. To overcome these issues, samples from individuals can be pooled together to gain a larger sample volume, but subsequently, information on the individual differences between patients is lost.

Over the years, different technologies including two-dimensional electrophoresis (2-DE), large-scale western blotting, and antibody microarrays have been used in proteomic analytics [25]. However, these methods come with their own limitations. Although 2-DE, which involves the separation of complex protein mixtures based on molecular charge and mass, provides information on the number of proteins and possible post-translational modifications, it lacks reproducibility and the ability to detect hydrophobic proteins and proteins with low abundance. For proteins with low abundance and small size, such as cytokines, antibody-based protein measurement techniques and multiplex immunoassay kits are commonly used. However, Western blotting and antibody microarray only identify proteins that have available antibodies, and therefore these methods are not suitable for the study of uncharacterized proteins. 

During the last decade, there have been significant improvements in mass spectrometry (MS), which can be implemented in the global analysis of all the proteins in a sample. These improvements include more advanced MS instruments, the added advantage of the hyphenating MS to separation techniques, e.g., liquid chromatography (LC), and the ability of tandem mass spectrometry (MS/MS) for peptide and protein structure elucidation. These advances mean that MS-based proteomics has become one of the most frequently chosen analytical methods for qualitative and quantitative investigation of complex protein samples [26,27] and it has enabled researchers to identify and quantify hundreds of proteins from tear samples as small as 1 μL [28]. Larger discovery studies performed with LC-MS/MS-based proteomics can thus produce enough data to perform further pathway analysis, helping researchers uncover the underlying biological function shifts.

One of the main pitfalls of the MS techniques in tear proteomics is connected to the fact that the aqueous layer contains several high abundance proteins, referred to as major tear proteins, including albumin (ALB), lysozyme (LYZ), and lactotransferrin (LTF). These high-abundance proteins result in a wide dynamic range of proteins, making the detection of lower abundance proteins challenging [25]. 

Other possible approaches to studying tears are also emerging. The study of microRNA [29,30] can provide a further understanding of the pathologies in ocular surface-associated tissues. Studies of small extracellular vesicles (sEVs) also enable further morphologic examination of the tear fluid [31]. Lipidomic and metabolomic tear analyses are also being carried out and produce complementary information about the tear fluid state and composition [4,21,32].

It is important to recognize that the selection of sampling methods, analytic technologies, and study population will have a great effect on the results. For the same reason, comparisons between studies can be challenging. For example, the age of the study population has been established to affect the tear protein levels [33]. Therefore, researchers must carefully record all information in their own work, ensure comparability between study groups, and interpret results with these differences in mind. Further details about the technical aspects of tear fluid proteomics can be found in our recent review [26].

In relation to glaucoma, the tear fluid is located far from the original site of damage, i.e., RGCs and optic nerve, but the tear fluid’s proximity to aqueous outflow channels, i.e., trabecular meshwork, Schlemm’s canal, and episcleral veins, could be beneficial as they are connected to the pathophysiological changes in glaucoma. In the following sections, we will also discuss how the tear fluid is closely connected to topical treatment effects as well as glaucoma surgery outcomes.

## 3. Glaucomatous Changes and Their Reflection on the Tear Fluid Proteomics

In order to understand the effects of individual tear proteins, it is important to understand how glaucomatous changes affect the tear fluid and other anterior parts of the eye. In many of the common glaucoma subtypes, as the normal aqueous humor outflow is disrupted, increased IOP and changes in the normal function of the eye can lead to biomechanical, vascular, and immune-related stress. This stress is thought to further lead to mitochondrial dysfunction, chronic oxidative stress, and the generation of reactive oxygen species (ROS), inducing RGC degeneration through inflammation, autophagy, and apoptotic pathways (Figure 1B) [34,35,36].

Tear proteomics makes it possible to non-invasively study, how the aforementioned disease mechanisms affect and reflect on the tear fluid and ocular surface. A previous study by Pieragostino et al. [37] examined the differences in tear protein levels between healthy and treatment-naïve primary open-angle glaucoma (POAG) glaucoma patients using LC-MS^E^ and identified 27 proteins, which differed in abundance between the study groups. The majority of the tear proteins, which differed significantly in this study, were upregulated among the glaucoma patients and included proteins associated with inflammatory response, free-radical scavenging, and cell-to-cell signaling and interaction. A similar study by Rossi et al. [38] examined the tear proteomics of treatment-naïve POAG patients and identified upregulation of ‘EV exosomes’ and, through the analysis of the EVs of the tear fluid, they identified that an inflammatory response is triggered through neutrophils in the glaucomatous ocular surface. These results indicate that the ocular surface and tear fluid could potentially reflect the glaucoma-induced changes, even before the diagnosis is formulated and treatment is started. The tear protein differences between controls and patients suffering from POAG and other glaucoma types (treatment-naïve and treated) [37,38,39,40,41,42,43,44,45,46,47,48,49,50] are reported and listed in Table 1.

Many of the proteins identified as up- or down-regulated with both treatment-naïve and treated glaucoma patients in Table 1 are well-known tear proteins associated with various OSDs, such as DE [6]. Furthermore, based on previously published results, the DE-associated up- and down-regulations of tear proteins resemble more the changes occurring among the treated glaucoma patients rather than the treatment-naïve glaucoma patients. For example, lysozyme (LYZ), a polymeric immunoglobulin receptor (PIGR), a prolactin-induced protein (PIP), and cystatin S (CST4), which have all been found to be decreased in DE [51], are observed to be increased in the treatment-naïve glaucoma patients, before decreasing in the topically treated patients. However, it is worth noting here that differences between DE and treated as well as treatment-naïve POAG patients have also been observed previously [47,48,49]. Results identified that POAG patients had increased levels of interleukin 6 (IL6), tumor necrosis factor (TNF) and vascular endothelial growth factor A (VEGFA), and decreased levels of interleukin 4 (IL4) in their tear fluid [47,48,49]. Therefore, the effects of DE and topical glaucoma medication-induced OSD on the ocular surface and tear fluid are not directly comparable conditions.

In addition to proteins, the level of homocysteine (Hcy), an amino acid, has been studied in tears in relation to glaucoma. Hcy is broken down by vitamins B6 and B12 and folic acid, and in the tear fluid, it has been identified to be elevated for both POAG and pseudoexfoliation glaucoma (PXG) patients [52,53]. The results indicate that issues in B-vitamin levels could be connected to glaucoma development/progression and clinical evidence supports this as well [54]. In fact, the establishment of the effects of vitamins is still largely ongoing as researchers attempt to discover which vitamins could help prevent or treat glaucoma [54,55].

Due to the myriad combinations of glaucoma types, treatment status, and medications in use, it has been difficult to establish sensitive and specific tear biomarkers which could detect glaucomatous changes at the early stages of the disease progression. Therefore, more work is needed in glaucoma-related tear biomarker discovery if they are to be used as a part of clinical glaucoma diagnostics. Although other sample materials such as aqueous humor or tissues do require invasive sampling techniques, they could provide complementary information about the health status elsewhere in the eye and provide a more comprehensive insight into glaucomatous effects taking place in the eye.

## 4. Tear Fluid Proteomics in the Evaluation of Therapeutic Outcomes in Glaucoma

### 4.1. Topical Medication

Since IOP is so far the only controllable pathophysiological mechanism of glaucoma, the treatment approaches focus on its lowering and control through increasing the outflow and decreasing the production of aqueous humor. It has been proven as an efficient treatment approach through multiple studies [56,57,58]. Due to lacking the means to regenerate the lost RGC and vision, early treatment is vital in the prevention of vision loss. The most common approach to treat glaucoma includes a selection of topical medications, i.e., ocular hypotensive drops, which patients often use for years and decades in different combinations due to the chronic nature of glaucoma.

Although the eye drops used to treat glaucoma are normally effective in lowering IOP, the prolonged use of topical glaucoma medications results in various changes in the anterior segments of the eye. These changes in the tissues include the loss of conjunctival goblet cells, Meibomian gland abnormalities, and structural changes to the ocular surface epithelium and they are often exacerbated by an increasing number of topical glaucoma medications as well as preservatives in the topical treatments [59,60,61]. In the tear fluid, these alterations can result in the thinning of the mucin [62] and lipid layers [63], tear fluid instability, and changes in the tear fluid composition [64]. For patients, these changes in the ocular surface tissue and tear fluid can be manifested through the irritation and inflammation of the ocular surface, and previous studies have indicated that glaucoma patients suffer more frequently from OSDs [65,66].

In addition to the active ingredients of the eye drops, preservatives used in eye drops, especially benzalkonium chloride (BAK), are shown to be a major cause of adverse reactions, and often a switch to preservative-free medication alleviates these symptoms [67,68,69,70]. Previous studies have highlighted that the prolonged use of these preservatives not only causes ocular surface inflammation but also induces oxidative stress in the tear film [71,72], further tipping off the balance of the ocular surface functions and increasing the risks of OSD development.

It is worth noting that the ocular surface effects can further differ depending not only on the active ingredients used in the topical treatment, which include, e.g., prostaglandin analogues, beta-blockers, carbonic anhydrase inhibitors, cholinergic compounds, alpha agonists, and rho kinase inhibitors, but also the type of glaucoma being treated. The large selection of active ingredients and preservatives as well as their use in combinations can make the study of disease mechanisms and treatment effects particularly difficult. Some previous studies examining the effects of topical glaucoma treatment have limited their focus to specific glaucoma subtypes, such as POAG and PXG, and specific topical treatment types to ensure more homogeneous groups to compare.

Pieragostino et al. [43] included both POAG and PXG patients who had been medically treated with latanoprost (a prostaglandin analogue) for at least 2 years. In the study, they identified various tear proteins, which differed in abundance in comparison to controls. For example, decreased levels of cystatins SN, SA, and S (CST1, CST2, and CST4) and increased levels of transferrin (TF) and S100 calcium-binding protein A4 (S100A4) are frequently encountered in DE patients’ tear fluid [6], and thus indicate worsened ocular surface health among the medicated POAG patients as well. Perhaps most interestingly, although the results indicated that both glaucoma subtypes’ tear fluid changes were connected to inflammatory pathways, distinct differences were observed; for example, S100A4 and S100 calcium-binding protein A8 (S100A8) were upregulated in POAG while CST2 and CST4 were upregulated in PXG patients. Based on previous studies on ocular surface pathologies, S100A4 and S100A8 have been reported to be increased in, e.g., conjunctivochalasis [73], while CST4 has been decreased in DE [7,51]. Based on these results, the differences between POAG and PXG are indicating that POAG patients’ ocular surface health becomes more compromised during treatment. However, similar findings were not identified at the cytokine level, based on a study by Vidal-Villegas et al. [74], as they did not report any statistically significant differences in tear fluid between POAG and PXG for the 27 cytokines measured in their study.

Another study with POAG patients [48] identified that IL6 and TNF were lowered in the tear fluid of POAG patients receiving oral supplements with antioxidants and fatty acids in addition to their normal hypertensive eye drops. These findings indicate that other treatment protocols are able to alter the tear protein levels and possibly alleviate the adverse effects caused by the topical treatment approaches.

In a study focusing on uveitic glaucoma patients, the treatment approaches (prostaglandin analogues only, corticosteroids only, prostaglandin analogues and corticosteroids, and no topical medication) were compared against each other [75]. In the case of uveitic glaucoma, the different treatments did not induce differing inflammation responses according to the tear cytokines measured in the study, including several interleukins (IL12p70, IL2, IL10, IL8, IL6, IL4, IL5, IL1B) and interferons alpha, beta, and gamma (IFNA, IFNB, IFNG).

Some glaucoma treatment studies have been less stringent with their inclusion criteria regarding either the glaucoma type or the active ingredients in their treatment. This way, more general findings of different effects of topical glaucoma treatment have also become available. Several cytokine studies have identified increased levels of many proinflammatory cytokines in tears of treated glaucoma patients, including interleukins (IL2, IL4, IL5, IL6, IL8, IL10, IL12, IL15, IL1B), TNF, C-C motif chemokine ligand 2 (CCL2), vascular endothelial growth factor (VEGF), fibroblast growth factor 2 (FGF2), and IFNG [47,76,77]. In studies focusing on prostaglandin analogue-treated glaucoma patients, IL1B, IL6, and matrix metallopeptidases 1, 3, and 9 (MMP1, MMP3, and MMP9) were seen to be increased, while TIMP metallopeptidase inhibitors 1 and 2 (TIMP1 and TIMP2) were decreased [78,79]. Instead of cytokine measurements, Wong et al. [80] performed a comparison using MS and discovered increased levels of S100A8, S100A9, secretoglobin family 2A member 1 (SCGB2A1), and tyrosine 3-monooxygenase/tryptophan 5-monooxygenase activation protein zeta (YWHAZ). Many of the findings listed here are also summarized in Table 2. Overall, increased levels of interleukins, metallopeptidases, and S100 proteins consistently indicate that prolonged use of topical glaucoma medications induces pro-inflammatory changes in the tear film, affects the extracellular matrix homeostasis, and deteriorates the ocular surface health.

In recent years, the study focus has shifted more towards the comparison of different treatment types. For example, a comparison between preserved and preservative-free eye drops is of interest now that the harmful effects of preservatives are better understood. Previous studies have examined the cytokine differences between patients receiving either preserved or preservative-free latanoprost medication. Martinez-de-la-Casa et al. [81] reported that several pro-inflammatory cytokines were upregulated among the preserved-treatment group, while glaucoma patients receiving preservative-free medication resembled the controls according to the tear cytokine levels. Manni et al. [82] observed increased levels of IL1B in the tears of glaucoma patients using preserved timolol. Reddy et al. [83] also reported increased MMP9 and decreased MMP2 in the tears of latanoprost-treated eyes when compared to bimatoprost. Based on these comparisons, it is possible to infer that the less harmful effects of milder and/or preservative-free eye drops are also detectable in the tear fluid.

Both Funke et al. [84] and Nättinen et al. [85] studied the tear proteomics changes of glaucoma patients who switched from preserved to preservative-free prostaglandin analogue. The study by Funke et al. [84] identified several pro-inflammatory proteins, including TF, complement C3, (C3), and cytokines, such as IL1β, IL2, and IL23α, which were increased among glaucoma patients using preserved eye drops in comparison to controls. Furthermore, 24 weeks after a switch to preservative-free medication, the levels of the identified proteins started to resemble the levels of the controls. In the latter study by Nättinen et al. [85], the glaucoma patients undergoing the switch were stratified and different responses to the preservative-free switch were identified. More specifically, glaucoma patients with more severe adverse effects appeared to benefit more from the switch. Overall, these tear proteomics studies do further support the clinical findings, which suggest that preservative-free medication spares the ocular surface from additional stress and inflammation frequently caused by the preserved medications.

### 4.2. Glaucoma Surgery

If the progression of glaucoma cannot be controlled with medication alone, or if the patient experiences adverse effects, difficulties, or unwillingness to perform topical therapy, more invasive methods, such as laser and incisional surgeries can be performed. Usually, glaucoma surgery is performed on patients with a long history of topical medication use, although in certain conditions, such as ACG and very high initial IOP, glaucoma surgery can be the first therapeutic choice.

A long history of continuous topical glaucoma medication prior to surgery, as well as the resulting ocular surface adverse reactions, have been found to negatively affect the ocular surgery outcome [65,86,87,88,89,90]. Other risk factors for glaucoma surgery failure include a high number of used topical glaucoma medications and preservatives as well as previous eye surgeries. In light of this information, emerging recommendations are suggesting preoperative outlines, which could be used to stabilize the ocular surface state prior to surgery. For example, a switch from preserved to preservative-free eye drops or an administration of artificial tears and preoperative anti-inflammatory drugs, e.g., fluorometholone, and management of lid hygiene could help reduce the ocular surface inflammation and increase the chances of a successful surgery [91].

There is a large variety of possible surgical techniques to choose from in glaucoma treatment. After Cairns introduced trabeculectomy in the 1960s [92], it has become the gold standard of glaucoma surgery and has long been the most popular surgical method for the treatment of glaucoma. It has been, however, challenged by the invention of surgical techniques using glaucoma implants [93] and non-penetrating glaucoma procedures such as deep sclerectomy [94]. All these techniques have been developed by modifying the surgical techniques, peri- or postoperative medication, or surgical implants.

The main goal of all these surgical techniques is to improve and redirect the aqueous outflow either to subconjunctival or suprachoroidal space. This can be achieved by creating a new pathway for aqueous humor to escape from the eye, either by using the eye’s own tissues or by inserting biocompatible tubes in the structures of the eye. The success of these operations is highly dependent on the controlled wound healing, which will enable a stable outflow of aqueous humor and a low but sufficient IOP since an exacerbated wound healing is the most important cause of surgical failure [95].

In relation to wound healing issues, it is further known that the preoperative state of the conjunctiva is one of the major risks leading to exacerbated wound healing and surgical failure, and for this reason, antifibrotic agents are frequently used in connection to the surgery [86,89]. Why wound healing differs between patients undergoing trabeculectomy is still unknown, although many risk factors have been identified, including younger age, African ancestry, higher baseline IOP, OSDs, previous ocular surgeries, as well as duration, frequency, and the number of topical glaucoma medications [89,91,96,97,98]. A better understanding of the individual clinical and biological factors affecting the surgery outcome is needed if we wish to identify efficiently the patients who are at risk of post-surgical adverse events.

In relation to tear fluid, previous clinical studies have found that postoperative tear fluid characteristics are associated with the morphologies of glaucoma filtering blebs created during filtration surgery [99]. It is hypothesized that the irregularities on the ocular surface caused by the bleb can affect the ocular surface health and indications of worsened ocular surface state have been observed in successfully operated patients at least 6 months after surgery [100]. Levels of proMMP2 and MMP2 activity have also been reported to be increased in both flat and cystic blebs in comparison to controls [101], indicating that postoperative alterations are manifested in the tear fluid composition as well.

Many of the previous tear proteomics studies have attempted to identify tear proteins, which could indicate surgical failure prior to surgery. Chong et al. [102] identified that CCL2 was not only increased in the tears of the topically medicated glaucoma patients in comparison to non-glaucomatous controls, but that patients with post-surgical failure within 6 months after surgery had higher levels of CCL2 at baseline in comparison to ‘successful’ glaucoma patients. Csősz et al. [103] reported a further 17 decreased and 6 increased tear proteins at baseline among the group with post-trabeculectomy complications. Many of the identified proteins were related to inflammatory and wound healing responses, indicating that inflammation also plays a role in the failure outcomes. A later study from the same group [104] also identified three cytokines, IFNγ, IL5, and colony-stimulating factor 2 (CSF2), which were expressed in lower levels in the failed outcome groups’ tears prior to surgery. However, these results do not explain failures associated with faster wound healing.

In addition to studies examining the potential predictive biomarkers for trabeculectomy failure, some studies have also extended their research to clinical variables and protein level follow-up. Burgos-Blasco et al. [105] identified that high levels of IL8 in tears could predict lower IOP reduction. A study by Vaajanen et al. [106], on the other hand, examined how the tear protein levels change after trabeculectomy. In this study, trabeculectomy patients were followed for 1 year after surgery and the results indicated an improvement in the ocular surface state both clinically and at the protein level. More specifically, conjunctival grading showed improved post-surgical conjunctiva state and, according to proteomics, the inflammatory responses in the tear fluid were reduced. These results identify the beneficial changes occurring as a result of a successful trabeculectomy and, perhaps more importantly, after topical medications are removed from the ocular surface. The findings of this study are supported by a previous study following the levels of oligomeric mucus/gel-forming mucin 5AC (MUC5AC) [107]. It was noted that although the levels of MUC5AC initially decreased after short-term medication and trabeculectomy, the levels were comparable to controls by the end of the study, 6 months after the operation.

Although many interesting studies have already examined the glaucoma surgery outcome biomarkers in tear proteomics, more research is needed to better understand the biological processes occurring during glaucoma operation failures. In order to achieve this, more longitudinal studies are necessary. Studies spanning over several years not only help us understand and stratify the surgery outcome failures further but also help us gain a deeper knowledge of the effects of prolonged use of topical glaucoma treatment and its cessation. The majority of the tear proteomics studies so far have focused on studying the effects and outcomes of the trabeculectomy method, and less attention has been paid to other surgical approaches. Therefore, studies focusing on other surgical methods, as well as comparisons between these methods, are needed to enable a more personalized treatment approach for glaucoma surgery patients.

## 5. New Approaches and the Future Directions of Tear Proteomics in Glaucoma Research

As is the case in many diseases, the current research aims to provide more targeted and personalized treatment options for patients in the future, and glaucoma is no different. In glaucoma, this is particularly needed due to the difficulties related to both topical medication and glaucoma surgery, such as high risks of adverse events and low compliance in topical treatments as well as surgical failures. Although the multifactorial nature of the disease is becoming better known, treatments still focus solely on the control of the IOP. A better understanding of the disease mechanisms at both molecular and pathway levels in different glaucoma subtypes can help in the development of the targeted treatment methods in the future.

Currently, potential diagnostic and therapeutic targets, i.e., biomarkers, have already been identified in various sample materials, including tears, but further development of these biomarkers into functional clinical tools is still in process. In addition, the attempts to discover a marker unique to, e.g., the topical treatment effects of glaucoma are still ongoing. Clinically available biomarkers could enable earlier detection of arising problems and the more straightforward initiation of medical therapy. In addition, a more individualized topical treatment selection would help reduce the risks of adverse effects, this way enhancing the compliance to treatment routine and patients’ well-being. Similar benefits would apply to glaucoma surgery as a mode of in-depth investigation of the ocular surface state prior to the surgery, and the assignment of personalized preoperative treatment could improve the chances of a successful outcome. During the surgery follow-up, the control of anti-inflammatory medication could be controlled based on the ocular surface state, and cascades leading to failure could be intervened early.

Although the recent evidence demonstrates the potential value of tear fluid proteomics in glaucoma diagnostics, certain confounding factors such as the effects of therapeutic interventions should be recalled when inspecting the pathogenic mechanisms and progression of the disease. Since the tear reflects more precisely the health and changes taking place in the ocular surface, it can be debated whether changes, which are initially thought to occur in the retina and optic nerve region, can be observed in tear fluid alone. Instead, it is clear that tear proteomics can be a vital tool in understanding the effects of topical medications, as these do largely affect the health of the ocular surface. In future studies, more can be achieved when tear proteomics is combined with information obtained from other sample materials as well as extensive clinical and lifestyle information. A recent study by Pinazo-Durán et al. [108] implemented computational analysis on a large collection of data originating from various imaging tools as well as enzyme linked immunosorbent assay (ELISA), LC, and Western blots. These types of analyses, which combine data from various sources, are likely to enable the discovery of the underlying biological pathways and associated molecules, which are most likely numerous in a multifactorial disease such as glaucoma.

## 6. Conclusions

The ocular surface and the dynamic role of tear fluid in regulating its sensitive homeostasis are directly affected by the medical and surgical interventions for glaucoma. Furthermore, tear fluid offers novel possibilities as a non-invasive liquid biopsy, which can enable the clinical diagnosis of various forms of glaucoma and the detection of the pathophysiologic mechanism involved in glaucoma development and progression. Although the history of tear fluid proteomics is relatively long, the recent development of modern technologies such as LC-MS/MS methods has enabled the production of proteomics data with hundreds of tear proteins and their associated abundance levels, enabling a more in-depth analysis of the biological functions and pathways underlying glaucoma. Together with an improved understanding of the role of the ocular surface in successful glaucoma care and rapid development of the medical and surgical therapeutic options, tear fluid proteomics offers a clinical tool to tailor the therapeutic strategies on individual bases. In the future, larger tear protein panels, possibly combined with other types of sample materials and powerful computational tools, could enable us to provide patients with more personalized treatment, all while also controlling the potential associated risks.

## Figures and Tables

**Table 1:** The glaucoma effects on tear proteins according to previous literature.

UniProt ID	Name	Symbol	Treatment-Naïve	Treated
P60709	Actin beta	ACTB	↑POAG [37]	
P63261	Actin gamma 1	ACTG1	↑POAG [37]	
P02768	Albumin	ALB	↑POAG [37]	
P04083	Annexin A1	ANXA1		↓POAG [43]
P25311	Alpha-2-glycoprotein, zinc-binding	AZGP1	↑POAG [37]	↓POAG [43]; ↓PXG [43]
P61769	Beta-2-microglobulin	B2M	↑POAG [37]	
P23560	Brain-derived neurotrophic factor	BDNF		↓NTG [44]
P37279	Cellular communication network factor 2	CCN2		↑PXG [45]; ↓PXF [45]
P26441	Ciliary neurotrophic factor	CNTF		↓POAG [46]
P20849	Collagen type IX alpha 1 chain	COL9A1	↑PXF [39]	
P01037	Cystatin SN	CST1		↓POAG [43]; ↓PXG [43]
P09228	Cystatin SA	CST2		↓POAG [43]; ↓PXG [43]
P01036	Cystatin S	CST4	↑POAG [37]	↓POAG [43]; ↓PXG [43]
P02751	Fibronectin 1	FN1	↓PXG [40]	
P04792	Heat shock protein family B (small) member 1	HSPB1	↑POAG [37]	
P01876	Immunoglobulin heavy constant alpha 1	IGHA1	↑POAG [37]	↓POAG [43]; ↓PXG [43]
P01877	Immunoglobulin heavy constant alpha 2	IGHA2	↑POAG [37]	↓POAG [43]
P01857	Immunoglobulin heavy constant gamma 1	IGHG1		↑POAG [43]
P01859	Immunoglobulin heavy constant gamma 2	IGHG2		↑POAG [43]
P01861	Immunoglobulin heavy constant gamma 4	IGHG4		↑POAG [43]
P01591	Joining chain of multimeric IgA and IgM	JCHAIN	↑POAG [37]	↓POAG [43]
P01834	Immunoglobulin kappa constant	IGKC	↑POAG [37]	↓POAG [43]
P37459	Interleukin12A	IL12A	↓POAG [41]	
P05231	Interleukin 6	IL6		↑POAG [47,48,49] *
P04264	Keratin 1	KRT1		↓POAG [43]; ↓PXG [43]
Q9GZZ8	Lacritin	LACRT		↓PXG [43]
P31025	Lipocalin 1	LCN1	↑POAG [37]	↓POAG [43]; ↓PXG [43]
P02788	Lactotransferrin	LTF	↑POAG [37]	
P61626	Lysozyme	LYZ	↑POAG [37]	↓POAG [43]; ↓PXG [43]
P03956	Matrix metallopeptidase 1	MMP1	↑PXG [40]; ↑PXF [40]	
P08253	Matrix metallopeptidase 2	MMP2	↑POAG [42]	
P14780	Matrix metallopeptidase 9	MMP9	↑POAG [42]; ↑PACG [42]; ↑PXF [40,42]	↑POAG [50]
P01840	Polymeric immunoglobulin receptor	PIGR	↑POAG [37]	↓POAG [43]
P12273	Prolactin induced protein	PIP	↑POAG [37]	↓POAG [43]; ↓PXG [43]
A5A3E0	POTE ankyrin domain family member F	POTEE/POTEF	↑POAG [37]	
Q6S8J3	POTE ankyrin domain family member E	POTEE/POTEF	↑POAG [37]	
P0CG38	POTE ankyrin domain family member I	POTEI	↑POAG [37]	
P0CG39	POTE ankyrin domain family member J	POTEJ	↑POAG [37]	
Q06830	Peroxiredoxin 1	PRDX1	↑POAG [37]	
Q99935	Opiorphin prepropeptide	OPRPN	↑POAG [37]	↓PXG [43]
Q16378	Proline rich 4	PRR4	↑POAG [37]	↓POAG [43]; ↓PXG [43]
P26447	S100 calcium-binding protein A4	S100A4		↑PXG [43]
O75556	Secretoglobin family 2A member 1	SCGB2A1		↓POAG [43]; ↓PXG [43]
P02787	Transferrin	TF	↑POAG [37]	↑PXG [43]
P01137	Transforming growth factor beta 1	TGFB1	↑PXG [40]; ↑PXF [40]	
Q96DA0	Zymogen granule protein 16B	ZG16B	↑POAG [37]	↓PXG [43]

POAG = primary open-angle glaucoma; PACG = primary angle-closure glaucoma; PXF = pseudoexfoliation syndrome; PXG = pseudoexfoliation glaucoma; NTG = normal-tension glaucoma; * = includes both treated and treatment-naïve.

**Table 2:** The effects of topical glaucoma medication on tear proteins according to previous literature.

UniProt ID	Name	Symbol	Tear Protein Changes for Glaucoma Patients Using…		
			Unspecified Topical Glaucoma Medication	Topical Glaucoma Medication with Prostaglandin Analogs	Topical Glaucoma Medication with Preservatives
P04083	Annexin A1	ANXA1		↓[43]	
P25311	Alpha-2-glycoprotein, zinc-binding	AZGP1		↓[43]	
P13500	C-C motif chemokine ligand 2	CCL2			↑[76]
P10147	C-C motif chemokine ligand 3	CCL3	↓[77]		
P01037	Cystatin SN	CST1		↓[43]	
P09228	Cystatin SA	CST2		↓[43]	
P01036	Cystatin S	CST4		↓[43]	
P09038	Fibroblast growth factor 2	FGF2	↑[77]		↑[81]
P09919	Colony stimulating factor 3	CSF3			↑[76]
P01876	Immunoglobulin heavy constant alpha 1	IGHA1		↓[43]	
P01877	Immunoglobulin heavy constant alpha 2	IGHA2		↓[43]	
P01857	Immunoglobulin heavy constant gamma 1	IGHG1		↑[43]	
P01859	Immunoglobulin heavy constant gamma 2	IGHG2		↑[43]	
P01861	Immunoglobulin heavy constant gamma 4	IGHG4		↑[43]	
P01834	Immunoglobulin kappa constant	IGKC		↓[43]	
P01591	Joining chain of multimeric IgA and IgM	JCHAIN		↓[43]	
P01579	Interferon gamma	IFNG			↑[76]
P01584	Interleukin 1 beta	IL1B		↑[78]	↑[76,82] *
P60568	Interleukin 2	IL2			↑[76,81]
P05112	Interleukin 4	IL4	↑[77]		↑[76]
P05113	Interleukin 5	IL5			↑[76,81]
P05231	Interleukin 6	IL6		↑[78]	↑[76]
P13232	Interleukin 7	IL7			↑[76]
P10145	Interleukin 8	IL8			↑[76]
P22301	Interleukin 10	IL10			↑[76,81]
P29459	Interleukin 12A	IL12A	↑[77]		↑[76,81]
P35225	Interleukin 13	IL13			↑[76,81]
P40933	Interleukin 15	IL15	↑[77]		↑[81]
Q16552	Interleukin 17A	IL17A			↑[76,81]
P04264	Keratin 1	KRT1		↓[43]	
Q9GZZ8	Lacritin	LACRT		↓[43]	
P31025	Lipocalin 1	LCN1		↓[43]	
P02788	Lactotransferrin	LTF		↓[43]	
P61626	Lysozyme	LYZ		↓[43]	
P03956	Matrix metallopeptidase 1	MMP1		↑[78]	
P08254	Matrix metallopeptidase 3	MMP3		↑[78]	
P14780	Matrix metallopeptidase 9	MMP9		↑[78,79]	↑[79]
Q99935	Opiorphin prepropeptide	OPRPN		↓[43]	
P01127	Platelet-derived growth factor subunit B	PDGFB			↑[81]
P01833	Polymeric immunoglobulin receptor	PIGR		↓[43]	
P12273	Prolactin induced protein	PIP		↓[43]	
Q16378	Proline rich 4	PRR4	↓[80]	↓[43]	
P26447	S100 calcium-binding protein A4	S100A4		↑[43]	
P05109	S100 calcium-binding protein A8	S100A8	↑[80]		
P06702	S100 calcium-binding protein A9	S100A9	↑[80]		
O75556	Secretoglobin family 2A member 1	SCGB2A1	↑[80]	↓[43]	
P02787	Transferrin	TF		↑[43]	
P01033	TIMP metallopeptidase inhibitor 1	TIMP1		↓[78]	
P16035	TIMP metallopeptidase inhibitor 2	TIMP2		↓[78]	
P01375	Tumor necrosis factor	TNF			↑[76,81]
P15692	Vascular endothelial growth factor A	VEGFA	↑[77]		
P63104	Tyrosine 3-monooxygenase/tryptophan 5-monooxygenase activation protein zeta	YWHAZ	↑[80]		
Q96DA0	Zymogen granule protein 16B	ZG16B		↓[43]	

Note, references can be in multiple columns. All comparisons were done against nonglaucomatous controls unless otherwise stated. * in comparison to preservative-free alternative.

## Data Availability

Not applicable.
